# Paraneoplastic Cerebellar Degeneration Revealing Non-Small Cell Lung Cancer: A Case Report

**DOI:** 10.7759/cureus.60258

**Published:** 2024-05-14

**Authors:** Badr Kharouaa, Latifa Boukhidous, Amine Hayoune, Sara Gartini, Hajar Charii, Meriem Rhazari, Afaf Thouil, Hatim Kouismi

**Affiliations:** 1 Department of Pulmonology, Faculty of Medicine and Pharmacy of Oujda, Mohammed VI University Hospital, Mohammed First University, Oujda, MAR; 2 Department of Pulmonology, Faculty of Medicine and Pharmacy of Oujda, Mohammed VI University Hospital, Mohammed first University, OUJDA, MAR; 3 Department of Pulmonology, Faculty of Medicine and Pharmacy of Oujda, Mohammed VI University Hospital, Mohammed first University, oujda, MAR; 4 Department of Pulmonology, Faculty of Medicine and Pharmacy of Oujda, Mohammed VI University Hospital, Mohammed first University, Oujda, MAR; 5 Department of Pulmonology, Research and Medical Sciences Laboratory, Faculty of Medicine and Pharmacy of Oujda, Mohammed VI University Hospital, Mohammed First University, Oujda, MAR

**Keywords:** large-cell, small-cell lung carcinoma, degeneration, paraneoplastic neurologic degeneration, subacute cerebellar degeneration

## Abstract

Paraneoplastic neurologic degeneration (PND) manifests as a sudden or subacute neurological syndrome often linked to underlying cancer, either overt or subclinical. Within the spectrum of PND, subacute paraneoplastic cerebellar degeneration (PCD) represents a distinctive subset. While rare, prompt diagnosis holds the potential to ameliorate both neurological and oncological outcomes. Herein, we present the case of a 61-year-old patient diagnosed with subacute cerebellar degeneration, ultimately unveiling non-small cell lung carcinoma.

## Introduction

Paraneoplastic neurologic degeneration (PND) encompasses a spectrum of rare manifestations observed in patients with various malignancies, including small cell lung cancer and neuroendocrine tumors [1]. Unlike metastatic, iatrogenic, toxic, or deficiency-related etiologies, PND arises from mechanisms often related to hormonal imbalances induced by active proteins or peptides secreted by malignant cells, as well as cytokines produced by tumor cells or the immune system [2].

One distinct manifestation within the realm of PND is paraneoplastic cerebellar degeneration (PCD), characterized by the onset of acute or subacute progressive pancerebellar dysfunction, typified by symptoms such as ataxia, dysarthria, and nystagmus [3]. This condition stems from immune-mediated injury targeting cerebellar Purkinje cells and is associated with various malignancies, with breast and pelvic tumors being the most commonly implicated [4]. Notably, PCD linked to lung cancer predominantly involves small-cell histology [5].

In this context, we present a diagnostic encounter with a 61-year-old male patient presenting with subacute cerebellar degeneration, ultimately attributable to non-small cell lung carcinoma. Through this case study, we aim to elucidate our diagnostic approach in such scenarios, shedding light on the clinical presentation, diagnostic challenges, and therapeutic implications associated with PCD secondary to lung malignancies.

## Case presentation

We present the case of a 63-year-old male, an active smoker for 25 pack years who occasionally consumed alcohol, and with no other past medical history, who presented to the emergency room with a two-day history of heaviness in his lower limbs, resulting in gait disturbance and walking difficulty, along with a balance disorder leading to recurrent falls.

Upon admission, the patient appeared alert and well-oriented in time and space, with a Glasgow Coma Scale (GCS) score of 15/15. He was afebrile, with a body temperature of 36.5°C, a heart rate of 80 beats per minute, blood pressure measuring 124/75 mmHg, respiratory rate of 19 breaths per minute, and oxygen saturation of 94% on ambient air.

Neurological examination revealed both static and kinetic cerebellar syndrome, characterized by dysarthria, diplopia, ataxic gait, peripheral neurogenic syndrome, muscle atrophy, and fasciculations of both lower limbs. No abnormalities were detected on the remainder of the physical examination.

Cerebrospinal fluid analysis results are detailed in Table 1, with no evidence of microbial growth observed in the culture.

**Table 1:** Laboratory findings. H: High.

Lab	Value	Reference range
Proteins	851 mg/L H	150 – 450 mg/L
Lymphocyte count	80 x 10^6/L H	< 5 x 10^6/L
Polymorphs	15 x 10^6/L H	< 1 x 10^6/L
Erythrocyte count	1251 x 10^6/L H	< 1 x 10^6/L

**Figure 1:**
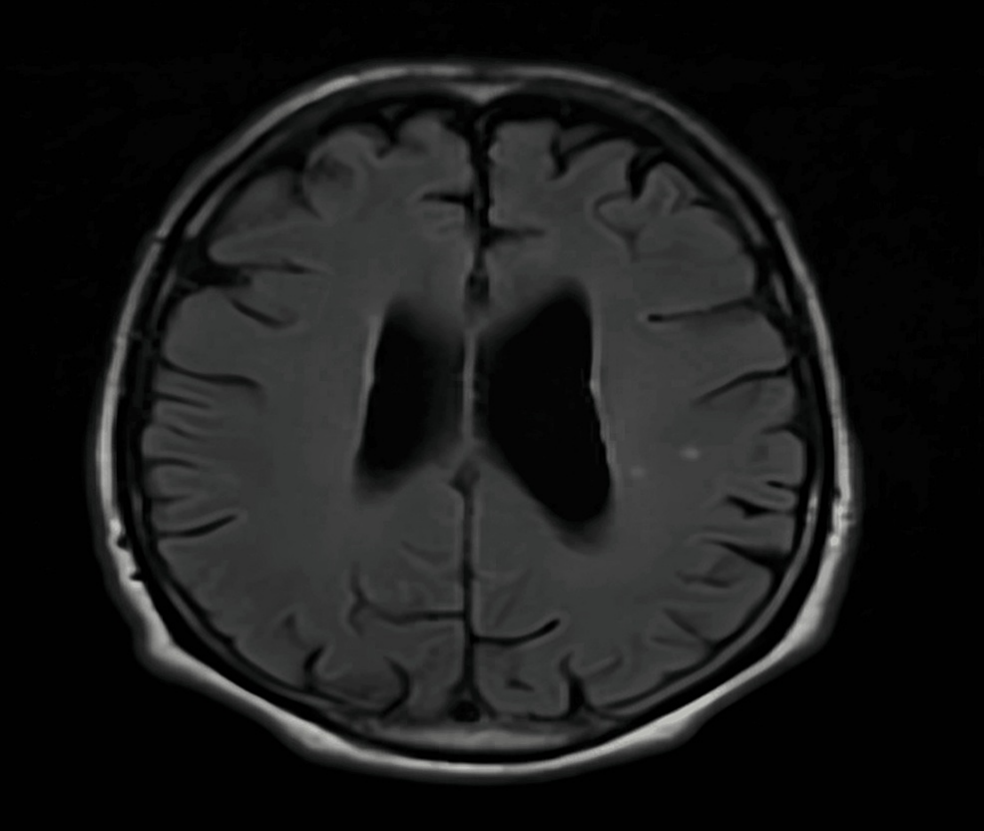
Brain MRI axial imaging without any remarkable results.

In consideration of these neurological findings, a brain MRI was conducted, yielding unremarkable results. This outcome served to rule out the presence of any expansive processes within the posterior cranial fossa, as depicted in Figure 1.

**Figure 2:**
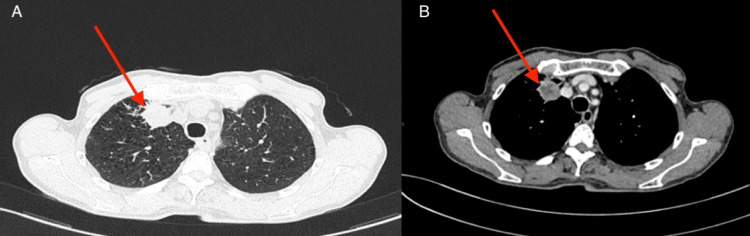
Axial chest CT scan; (A) lung window and (B) mediastinal window demonstrating a well-circumscribed, anterior mass located in the ventral segment of the right upper lobe of the lung (red arrows).

Following the initial assessments, which included the evaluation of onco-neuronal antibodies (Hu, Ri, Yo, CV2, amphiphysin, and Ma2), all of which returned negative results, as well as normal levels of vitamin E, B12, and thyroid hormones, diagnostic uncertainty persisted. Given the absence of clear diagnostic leads from these initial investigations, a high suspicion of an underlying malignancy with paraneoplastic origin was raised. Subsequently, a CT scan of the thorax, abdomen, and pelvis, with and without contrast injection (Figure 2), was conducted. The imaging revealed a sizable, well-defined anterior mass located in the ventral segment of the right upper lobe of the lung.

**Figure 3:**
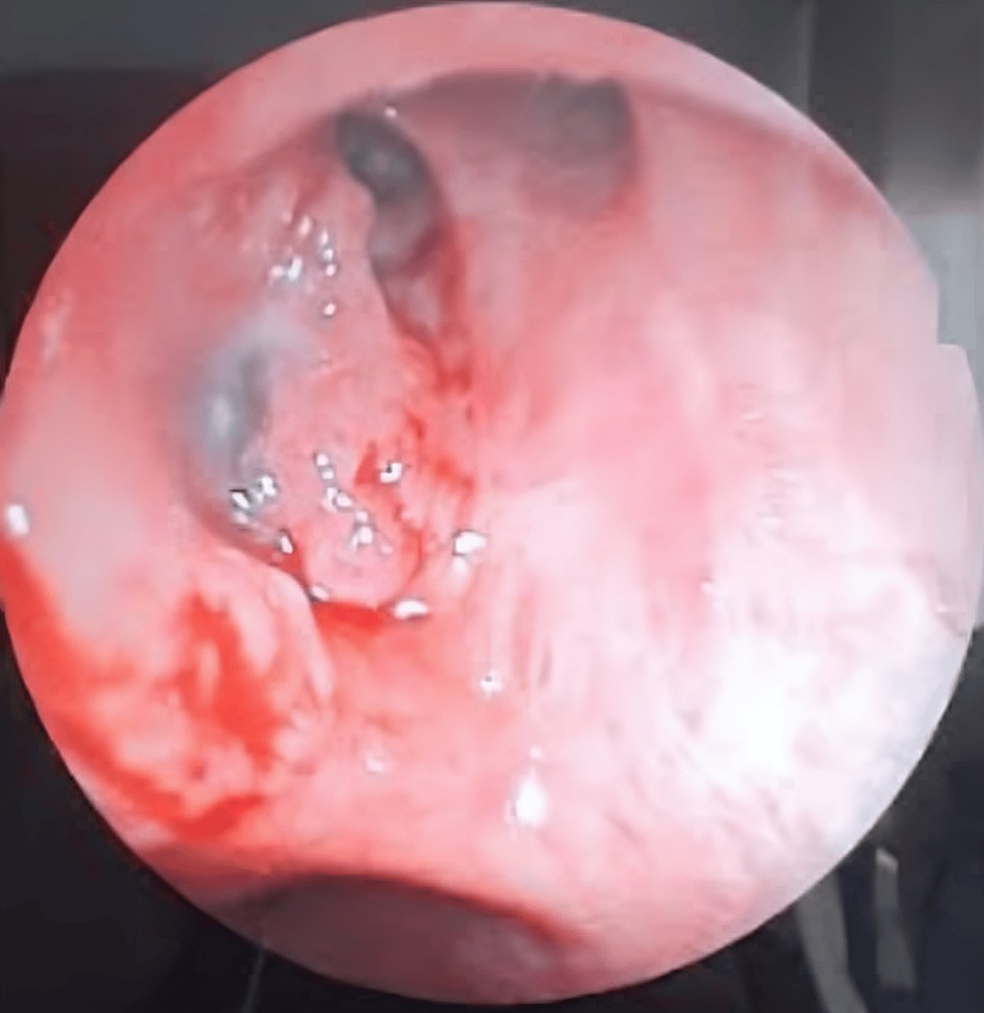
Bronchoscopic appearance shows the thickening of the right upper inter-lobar spurs with an irregular mucosa.

**Figure 4:**
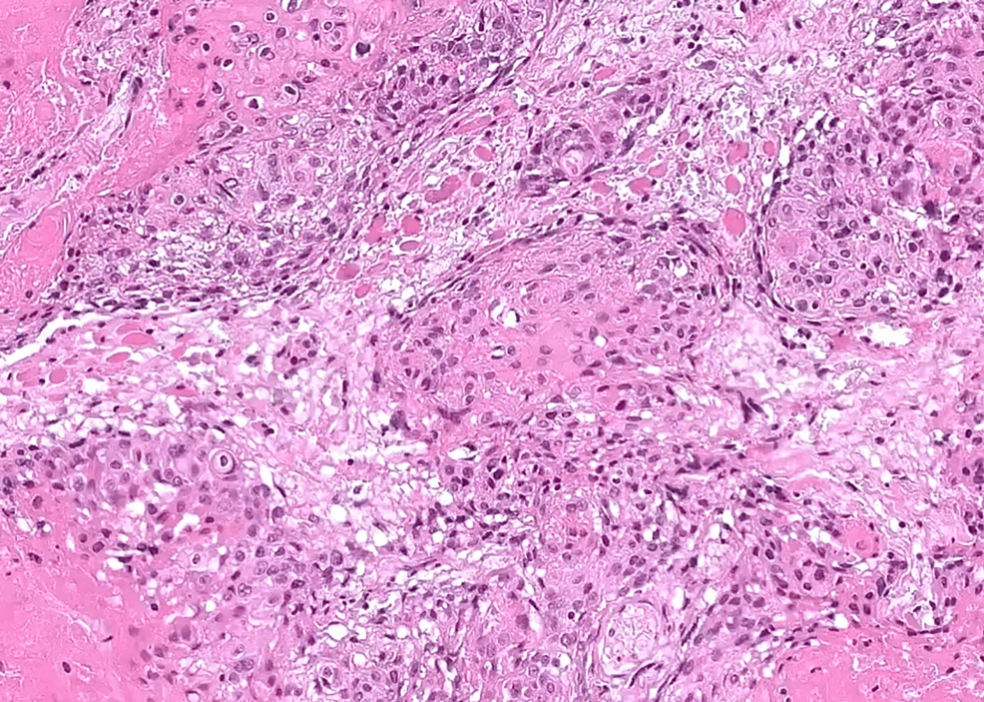
Histomorphological examination of the biopsy reveals a proliferation composed of polygonal cells with large, irregular nuclei and abundant eosinophilic cytoplasm. Nests of keratinisation are also present (H&E, x200). H&E: Hematoxylin and eosin.

A bronchoscopy with biopsy was performed and showed a thickening of the right upper inter-lobar spurs with an irregular mucosa (Figure 3). The histological examination unveiled features consistent with a non-small cell carcinoma, further characterized by an immunohistochemical profile indicating a squamous cell carcinoma subtype, with negative staining for TTF1 and CK7, and positive staining for p63 (Figure 4).

Consequently, the decision was made to initiate treatment with methylprednisolone bolus (500 mg/day for three days), followed by oral methylprednisolone therapy (2 mg/kg/day) for a duration of 15 days. However, despite these therapeutic measures, no clinical improvement was observed.

Subsequently, the patient was referred to the oncology department for palliative care in light of his progressive clinical deterioration. Unfortunately, despite medical intervention, the patient succumbed to the illness one month following the diagnosis.

## Discussion

PCD is predominantly associated with lung and gynecological malignancies, affecting both sexes. The onset of PCD typically mirrors the age distribution of cancer diagnoses [6]. Clinical manifestations of PCD may manifest at varying intervals relative to the primary tumor, spanning from several months to years, and can serve as an indicator for tumor detection. Symptoms commonly include bilateral static and kinetic cerebellar syndrome, dysarthria, as well as nystagmus and vertiginous syndrome [7]. In the case presented herein, neurological symptoms served as the initial manifestation of the underlying tumor.

In cases where a paraneoplastic etiology is suspected, clinical management necessitates a sequential radiological approach, beginning with a computed tomography of the chest, abdomen, and pelvis (CT CAP), followed by positron emission tomography (PET) imaging if indicated. It is paramount to underscore the imperative of diligently investigating any potential underlying malignancy. Initial radiological assessments frequently yield unremarkable findings, with subsequent imaging over prolonged intervals potentially elucidating characteristic signs such as cerebellar atrophy and dilation of the fourth ventricle, often with sparing involvement of the brainstem [8,9]. Notably, in the present case, MRI proved inconclusive in detecting any discernible abnormalities.

The pathophysiology underlying PCD remains inadequately elucidated, though autoimmune mechanisms have been proposed. It is hypothesized that PCD arises from a phenomenon of molecular mimicry, wherein the tumor aberrantly expresses proteins normally present in the nervous system, thereby triggering an autoimmune response. A defining feature of these syndromes is the presence of circulating autoantibodies, detectable in both serum and cerebrospinal fluid (CSF), with a notable association with specific neuronal nuclear proteins (SNPs) [9,10]. Among these autoantibodies, those targeting intracellular (onconeural) or membrane-bound epitopes have been identified, including Hu, Yo, CV2, Ri, Ma, Tr/DNER, amphiphysin, and Sox1 [10].

The therapeutic approach to PCD primarily revolves around the management of the underlying malignancy. However, conventional immunosuppressive or immunomodulatory agents have demonstrated limited efficacy in this context [8]. Despite appropriate anti-tumor interventions, the prognosis of PCD remains guarded, often contingent upon the persistence of clinical manifestations [10,11]. In the case described here, the patient underwent systemic corticosteroid therapy for PCD, yet failed to exhibit significant improvement, with a notable decline in his overall condition. Tragically, he succumbed to the illness one month following diagnosis.

## Conclusions

In conclusion, paraneoplastic cerebellar degeneration (PCD) represents a challenging clinical entity characterized by its association with various malignancies and its debilitating neurological manifestations. Despite advancements in understanding its pathophysiological basis, therapeutic options remain limited, with the primary focus centered on treating the underlying tumor. The case presented underscores the diagnostic complexities and therapeutic challenges inherent in managing PCD, as exemplified by the lack of clinical improvement despite aggressive treatment measures. Given the multifaceted nature of this condition, frequent follow-up evaluations are imperative, particularly when the suspicion of a paraneoplastic etiology arises. Moving forward, further research efforts are warranted to unravel the intricate mechanisms underlying PCD and to explore novel therapeutic avenues aimed at improving outcomes for affected individuals. Ultimately, enhancing our understanding of PCD holds the promise of refining diagnostic strategies, optimizing treatment approaches, and ultimately improving the prognosis for patients afflicted by this devastating condition.

## References

[1] Zekeridou A, Majed M, Heliopoulos I, Lennon VA (2019). Paraneoplastic autoimmunity and small-cell lung cancer: neurological and serological accompaniments. Thorac Cancer.

[2] Pelosof LC, Gerber DE (2010). Paraneoplastic syndromes: an approach to diagnosis and treatment. Mayo Clin Proc.

[3] Peterson K, Rosenblum M K, Kotanides H, Posner JB (2011). Paraneoplastic cerebellar degeneration: I. A clinical analysis of 55 anti-Yo antibody-positive patients. Neurology.

[4] Fanous I, Dillon P (2015). Paraneoplastic neurological complications of breast cancer. Exp Hematol Oncol.

[5] Konishi J, Yamazaki K, Chikai K (2004). Paraneoplastic cerebellar degeneration (PCD) associated with squamous cell carcinoma of the lung. Intern Med.

[6] Gatti G, Simsek S, Kurne A (2003). Paraneoplastic neurological disorders in breast cancer. Breast.

[7] Rojas-Marcos I, Rousseau A, Keime-Guibert F, Reñé R, Cartalat-Carel S, Delattre JY, Graus F (2003). Spectrum of paraneoplastic neurologic disorders in women with breast and gynecologic cancer. Medicine (Baltimore).

[8] Honnorat J, Cartalat-Carel S, Ricard D (2009). Onco-neural antibodies and tumour type determine survival and neurological symptoms in paraneoplastic neurological syndromes with Hu or CV2/CRMP5 antibodies. J Neurol Neurosurg Psychiatry.

[9] Shams'ili S, Grefkens J, de Leeuw B (2003). Paraneoplastic cerebellar degeneration associated with antineuronal antibodies: analysis of 50 patients. Brain.

[10] Viaccoz A, Honnorat J (2012). Évolutions conceptuelles des syndromes neurologiques paranéoplasiques (Article in French). Prat Neurol - FMC.

[11] Noorani A, Sadiq Z, Minakaran N, Coleman C, Thomas VA, Mokbel K (2008). Paraneoplastic cerebellar degeneration as a presentation of breast cancer - a case report and review of the literature. Int Semin Surg Oncol.

